# Variation of the Phytochemical Constituents and Antioxidant Activities of *Zingiber officinale* var. *rubrum* Theilade Associated with Different Drying Methods and Polyphenol Oxidase Activity

**DOI:** 10.3390/molecules21060780

**Published:** 2016-06-17

**Authors:** Ali Ghasemzadeh, Hawa Z. E. Jaafar, Asmah Rahmat

**Affiliations:** 1 Department of Crop Science, Faculty of Agriculture, Universiti Putra Malaysia, 43400 Serdang, Selangor, Malaysia; hawazej@upm.edu.my; 2 Department of Nutrition & Dietetics, Faculty of Medicine & Health Sciences, Universiti Putra Malaysia, 43400 Serdang, Selangor, Malaysia; profasmah@gmail.com

**Keywords:** *Zingiber officinale* var. *rubrum* Theilade, storage, phytochemicals, polyphenol oxidase, DPPH activity, FRAP activity

## Abstract

The effects of different drying methods (freeze drying, vacuum oven drying, and shade drying) on the phytochemical constituents associated with the antioxidant activities of *Z. officinale* var. *rubrum* Theilade were evaluated to determine the optimal drying process for these rhizomes. Total flavonoid content (TFC), total phenolic content (TPC), and polyphenol oxidase (PPO) activity were measured using the spectrophotometric method. Individual phenolic acids and flavonoids, 6- and 8-gingerol and shogaol were identified by ultra-high performance liquid chromatography method. Ferric reducing antioxidant potential (FRAP) and 1,1-diphenyl-2-picrylhydrazyl (DPPH) assays were used for the evaluation of antioxidant activities. The highest reduction in moisture content was observed after freeze drying (82.97%), followed by vacuum oven drying (80.43%) and shade drying (72.65%). The highest TPC, TFC, and 6- and 8-shogaol contents were observed in samples dried by the vacuum oven drying method compared to other drying methods. The highest content of 6- and 8-gingerol was observed after freeze drying, followed by vacuum oven drying and shade drying methods. Fresh samples had the highest PPO activity and lowest content of flavonoid and phenolic acid compounds compared to dried samples. Rhizomes dried by the vacuum oven drying method represent the highest DPPH (52.9%) and FRAP activities (566.5 μM of Fe (II)/g DM), followed by freeze drying (48.3% and 527.1 μM of Fe (II)/g DM, respectively) and shade drying methods (37.64% and 471.8 μM of Fe (II)/g DM, respectively) with IC_50_ values of 27.2, 29.1, and 34.8 μg/mL, respectively. Negative and significant correlations were observed between PPO and antioxidant activity of rhizomes. Vacuum oven dried rhizomes can be utilized as an ingredient for the development of value-added food products as they contain high contents of phytochemicals with valuable antioxidant potential.

## 1. Introduction

Herbs and spices produce a variety of phytochemicals or secondary metabolites and have been used not only as food preservatives and flavourings, but also as traditional medicines for thousands of years. Today, a valuable line of study in the medical industry is appraising phytochemicals to ascertain whether they have any biological activities with potential benefits to human health. Zingiberaceae plants have received much attention because they produce various compounds that are useful in cooking, such as spices and herbs, seasoning and flavouring agents, and in the medicinal and cosmetics industries as antimicrobial and antioxidant agents [1,2,3]. *Zingiber officinale* var. *rubrum* Theilade, locally known in Malaysia as Halia bara, is a widely used spice in Asia. Today, ginger is highly valued for its medicinal properties and it shows an important role in human primary health care worldwide. Ginger contains various phytochemicals and biologically active compounds such as phenolics, flavonoids, 6-gingerol, 6-shogaol, and zingerone [4,5]. From among the identified components, shogaols and gingerols were found to be the most abundant bioactive compounds in ginger, having numerous pharmacological benefits, including antioxidant, analgesic, anticancer, antipyretic, and anti-inflammatory properties [6,7,8]. The drying, extraction, storage, and processing steps have a significant impact on the quality of the herbs [9]. Herbal materials must be dried under specified conditions in order to retain their nutritional quality and avoid contamination and deterioration, while medicinal herbs should be dried to protect their phytochemical efficiency [10]. Furthermore, drying also reduces the cost of the final product, as transportation and storage costs are typically determined by product weight. However, the stability of phytochemicals, especially phenolics, are influenced by several factors [11]. Among them, polyphenol oxidase (PPO) plays an important role in the degradation of phenolics and flavonoids of crops. Catalysis of phenolic compounds by PPO has been reported in previous studies [12]. PPO showed variable activity under different storage conditions and its activity is dependent on the drying method of samples, storage, and processing conditions [13,14]. Farmers tend to store rhizomes in underground tunnels, where the maintenance of optimum humidity and temperature control are impossible, and it is difficult and expensive to preserve the ideal drying and storage conditions for ginger rhizomes. This results in spoilage and the ginger roots sprout after a few months. Developing a method for the drying of harvested ginger roots, without any loss in the quality, is therefore very important to ginger growers, ginger-processing personnel, and perennial consumers. Currently, there is little information on the drying of *Z. officinale* var. *rubrum* rhizomes in Malaysia. In addition, little is known about the degradation phenolics and flavonoids produced by PPO activities associated with the drying method. The aim of this study was to evaluate the effect of different drying methods on the phytochemical constituents (TPC, TFC, and individual phenolics and flavonoids, 6- and 8-gingerol and shogaol) and changes in antioxidant activities of *Z. officinale* var. *rubrum* in relation to PPO activity*.* The results of this study should provide useful information about the rhizome drying process of *Z. officinale* var. *rubrum*, thereby preserving food quality and bioactivity by avoiding the degradation of phytochemicals.

## 2. Results and Discussion

### 2.1. Moisture Content

**Figure 1:**
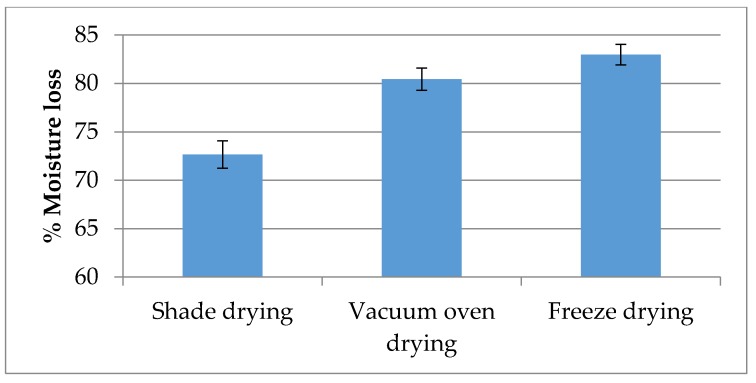
Effect of different drying methods on moisture loss in *Z. officinale* var. *rubrum* Theilade. Bars represent standard error of the means.

Percentages of moisture losses are shown in Figure 1. Significant differences were observed between drying methods for moisture content. The highest reduction in moisture content was observed under freeze drying (82.97%), followed by vacuum drying (80.43%) and shade drying (72.65%) methods. Moisture loss may affect organoleptic parameters in herbs such as colour and taste.

### 2.2. Total Flavonoid and Phenolic Content

Significant differences were observed between drying methods for TPC and TFC. Highest TPC was observed in the vacuum oven drying method (18.44 milligram gallic acid equivalents (GAE)/g dry material) followed by freeze drying (13.49 mg GAE/g DM) and shade drying (10.7744 mg GAE/g DM). Vacuum oven drying showed the highest TFC (8.27 milligram quercetin equivalents (QE)/g DM) followed by freeze drying (6.44 mg QE/g DM) and shade drying (4.93 mg QE/g DM). Fresh rhizomes represent the lowest TPC (7.58 mg GAE/g DM) and TFC (3.65 mg QE/g DM) compared to dried samples. Shade drying of ginger rhizomes resulted in significant losses in TFC and TPC compared to other drying methods. It has been reported that the amount of phytochemicals in dried samples is higher than in fresh samples because in fresh samples enzymatic actions are able to degrade these bioactive compounds [15]. Previous studies have shown that oven drying of some herbs resulted in the degradation of some metabolites [16,17]. The results of the present study were consistent with a previous studies which reported that air-dried marionberry (*Rubus fruticosus*), strawberry (*Fragaria ananassa*), corn (*Zea mays*) [18] and wild cosmos (*Cosmos caudatus*) [19] yielded lower TPC than freeze dried samples. However, it is difficult to recommend a common method for drying herbal materials, as the effect of the drying method varies in different herbs and is dependent on the species and type of metabolites they contain. Some herbs need to be dried at low temperatures, while others require drying at high temperatures.

### 2.3. Content of 6- and 8-gingerol and shogaol

The contents of 6- and 8-gingerol and shogaol were identified in ginger rhizomes and the data are shown in Table 1, with significant differences observed between the different drying methods. The highest contents of 6- and 8-gingerol were observed in the freeze drying method (5.82 and 3.58 mg/g DM, respectively), followed by vacuum oven drying (4.61 and 3.42 mg/g DM, respectively) and shade drying (3.79 and 2.84 mg/g DM, respectively) methods. No significant differences were observed between freeze and vacuum oven drying for 8-gingerol content. Fresh samples showed the lowest content of 6-gingerol (3.14 mg/g DM) and 8-gingerol (2.50 mg/g DM) compared to dried samples.

Vacuum oven drying represents the highest content of 6- and 8-shogaol (7.49 and 5.73 mg/g DM, respectively) followed by freeze drying (4.66 and 4.19 mg/g DM, respectively) and shade drying (4.21 and 3.80 mg/g DM, respectively) methods. The lowest contents of 6- and 8-shogaol were observed in fresh samples (2.20 and 2.12 mg/g DM, respectively). Fresh samples showed the highest content of gingerol compounds compared to shogaol compounds, while after drying the samples. The amount of shogaols increased compared to the gingerol compound. Freeze drying is a cold drying method, whereas vacuum oven drying is a hot drying method. The results indicated that with an increase in the drying temperature, the 6- and 8-shogaol contents increased in the rhizomes. This could be due to hydrolysis of gingerol to shogaol at high temperatures, which has been reported in previous studies [20].

### 2.4. Individual Flavonoids and Phenolics

In this study, five flavonoids and five phenolic acids were identified in the samples. As can be seen from the data in Table 2, significant differences were observed between the different drying methods in individual flavonoid and phenolic acid content.

Ginger rhizomes showed the highest contents of epicatechin, catechin, quercetin, rutin, ferulic, and cinnamic acid when dried with the vacuum oven drying method, followed by the freeze and shade drying methods. However, the highest contents of kaempferol, tannic, and syringic acid were observed in ginger rhizomes that were dried using the freeze drying method, followed by vacuum oven drying and shade drying methods. No significant difference was observed between vacuum oven drying and freeze drying methods for gallic acid content. Fresh samples represent the lowest content of flavonoid and phenolic acid compounds compared to dried samples. Freeze drying resulted in slight but significant declines in flavonoid and phenolic acid contents compared to vacuum oven drying. Cinnamic and syringic acid were not detected in fresh samples. In *Z. officinale* var. *rubrum,* quercetin and gallic acid were the most abundant flavonoid and phenolic acid compounds, respectively. Among the phenolic acids identified, the most abundant according to the highest concentration were as follows: gallic acid > ferulic acid > tannic acid > cinnamic acid > syringic acid. Generally, in the food industry drying treatment is completed in order to preserve the food samples through the removal of moisture/water. If the drying conditions are not appropriate degradation of some phytochemicals can occur. The relative instability of some flavonoids and phenolic compounds in samples may indicate a sensitivity of these compounds to different drying treatments [21]. However, under some circumstances, drying has advantages in protecting the phytochemicals and may not decrease their health beneficial quality. For instance, natural antioxidants, such as quercetin [22], were reported to possess a latent stability to drying processes.

### 2.5. Antioxidant Activity

The results of antioxidant activity of *Z. officinale* var. *rubrum* rhizomes dried with different methods and tested with DPPH and FRAP assays are shown in Table 3; these data show that rhizomes dried using the vacuum oven drying method showed the highest DPPH (52.9%) and FRAP activities (566.5 μM of Fe (II)/g DM) followed by freeze drying (48.3% and 527.1 μM of Fe (II)/g DM, respectively) and shade drying (37.64% and 471.8 μM of Fe (II)/g DM, respectively). All samples showed lower DPPH and FRAP activity compared to quercetin and gallic acid (positive controls). Fresh samples represent the lowest DPPPH (22.5%) and FRAP activities (348.8 μM of Fe (II)/g DM) compared to dried samples. The IC_50_ represents the half maximal inhibitory concentration of *Z. officinale* var. *rubrum* rhizome extract. A lower IC_50_ indicates a stronger free radical inhibition (strong free radical inhibitors are active at low concentrations). In this study, lower IC_50_ values were observed in rhizomes dried with vacuum oven drying (27.2 μg/mL), followed by freeze drying (29.1 μg/mL) and shade drying (34.8 μg/mL). The IC_50_ values of all samples were higher than quercetin and gallic acid (positive controls). Freeze dried rhizomes of *Z. officinale* var. *rubrum* had the least decline in antioxidant activities using both the DPPH and FRAP assays compared with rhizomes dried using the vacuum oven drying method.

The results from previous studies indicate that antioxidant activities of herbs/crops are associated with phytochemical content [23,24]. In the current study, the higher antioxidant activity of *Z. officinale* var. *rubrum* rhizomes dried using the vacuum oven method could be attributed to the high content of flavonoids, phenolics, or 6- and 8-gingerol content. An increase in antioxidant activity following thermal treatment has been reported in tomatoes [25], sweet corn [26], shiitake mushrooms [27], and ginseng [28]. An increase in antioxidant activity in samples that are dried at higher temperatures could be due to the release of bound phenolic compounds brought about by the breakdown of cellular constituents and the formation of new compounds with enhanced antioxidant potential [29]. Declines in antioxidant activity resulting from shade drying could be attributed to enzymatic degradation because the drying process was completed at room temperature and it took several days for samples to dry. Contrary to our results, shade drying of lemon balm, oregano, and peppermint at 25–32 °C for 10 days showed variable antioxidant activities that ranged from significant increases to significant decreases [30]. It has been reported that drying of nutmeg (*Myristica fragrans*) using a hot air drying method resulted in an increase in safrole and myristicin content and could be associated with increased free radical scavenging activity [29].

### 2.6. Polyphenol Oxidase Activity

**Figure 2:**
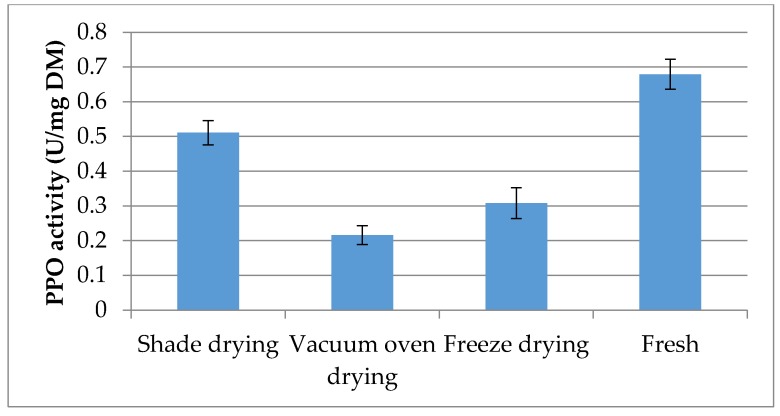
Effect of different drying methods on PPO activity in *Z. officinale* var. *rubrum* Theilade. Bars represent standard error of the means.

Enzymatic browning is one of the significant problems in number of crops and fruits, usually resulting in negative effects on colour, taste and flavor. The reaction is due mainly to oxidation of polyphenol compounds by polyphenol oxidase. Figure 2 shows the results of PPO activity in rhizomes dried with the three different methods as well as fresh rhizomes. Significant differences were observed between drying methods for PPO activity. Highest PPO activity was observed in fresh samples (0.679 U/mg DM) followed by shade (0.511 U/mg DM), freeze (0.308 U/mg DM), and vacuum oven (0.216 U/mg DM) drying methods. In the present study, fresh samples represent the lowest content of phytochemicals and antioxidant activity compared to dried samples. This could be due to inactivation of deteriorative enzymes such as lipoxygenase and PPO by the drying process. PPO, mixtures of monophenol oxidase, and catechol oxidase enzymes are widely present in plant tissues and can oxidise diphenols in the presence of oxygen molecules, causing rapid enzymatic oxidation of natural antioxidants. PPO is reported to be completely inactivated by the drying process [31]. Lim and Murtijaya [21] suggested that inactivation of PPO may be a reason why dried herbs can have higher secondary metabolites than fresh samples. Lower TPC and TFC in fresh samples are probably due to the oxidation by PPO. Oxidation of quercetin by PPO has been reported previously [32].

### 2.7. Correlation between Antioxidant Activities and Phytochemical Content

It is important to examine the correlation between phytochemical content and biological activity of crops and herbs to determine the corresponding compounds for biological activity in each plant. Such information could help researchers establish suitable conditions or to use suitable techniques in order to enhance these highlighted compounds. Thus, it is important to understand which antioxidant activity of *Z. officinale* var. *rubrum* rhizome is associated most with which compound. To this end, we performed correlation analysis for antioxidant activities and identified compounds (Table 4). Table 4 shows that all identified compounds had positive and significant (*p* < 0.05) correlations with DPPH and FRAP activity in ginger extracts. According to the obtained results, DPPH and FRAP activities are mostly associated with quercetin, 6-, and 8-shogaol contents. Positive and significant correlation between polyphenolic compounds and antioxidant activity of herbs and spices has been reported previously [33,34,35]. Based on this correlation analysis, it appears that quercetin (R^2^ = 0.940), and 6- and 8-shogaol (R^2^ = 0.942, 0.944, respectively) are corresponding compounds in *Z. officinale* var. *rubrum* rhizome extracts for free radical scavenging power. Antioxidant activities were significantly negatively correlated with PPO activity (R^2^_DPPH_: −0.911 and R^2^_FRAP_: −0.884). On the other hand, with increasing PPO activity, oxidation of phenolic compounds was enhanced in samples, and following that, antioxidant activity of samples decreased. Negative and significant correlation between PPO activity and TPC has been reported in a recent study [36].

## 3. Materials and Methods

### 3.1. Plant Sampling

Rhizomes of *Z. officinale* var. *rubrum* Theilade (Halia bara) were collected from a village in Bentong, Pahang, Malaysia. Rhizomes were washed with pure water and soaked for 30 min in a solution of mancozeb (0.3%). The rhizomes were cut into 3–5 cm pieces containing 2 to 3 buds. Then the rhizomes were germinated for two weeks in 15 × 15 cm growing pots filled with peat moss (each weighed about 1 kg). The rhizomes were planted 6 cm deep into the peat moss with the buds facing upward. The rhizomes were germinated under glasshouse condition. After two weeks, when the young leaves of seedlings reached 5 cm of height the seedlings were transplanted into 45 × 38 cm polyethylene bags which were to be filled with a soil-less mixture containing burnt rice husk and coco peat in a 1:1 ratio (each weighed about 6 kg). The described preparation was carried out at the glasshouse complex of University Putra Malaysia (UPM). The mean daily temperature 30 °C, mean relative humidity 70%–80% and highest irradiance level at 1650 μmol/m^2^/s and whilst minimum at 44 μmol/m^2^/sec. Harvesting was done at 9 months after plantation. Rhizome, leaf and stem were separated and washed with pure water. Samples were submitted to the Institute of Bioscience (Universiti Putra Malaysia) and were identified as *Zingiber officinale* var. *rubrum* Theilade with voucher specimen of SK 3422/12.

### 3.2. Drying of Fresh Ginger

The fresh harvested ginger rhizomes (200 g) were dried using the following drying methods: (1) drying in the shade at ambient temperature in a dark well-ventilated room at mean temperature of 15 °C and mean humidity of 50% for two weeks; (2) drying in a vacuum oven at 45 °C for 72 h in a vacuum of 600 mbr) freeze drying in a freeze drier at a temperature of −60 °C, pressure of 0.071 mbar for 72 h (samples were placed in the −80 °C freezer for one day before lyophilisation). Fresh samples were considered the control. Drying was performed in six replicates for each method and all the dried samples were kept in aluminium containers and stored at −20 °C prior to extraction and future analysis. Moisture content of samples was measured by using the following formula:

%Moisture = [(fresh weight − dry weight)/fresh weight] × 100
(1)

### 3.3. Extraction

Dried and fresh rhizomes (5 g) were extracted with ethanol (100 mL). Extraction was performed after refluxing at 65 °C for 35 min, after which the solutions were cooled at room temperature and filtered through Whatman filter paper (No. 1). Excess solvent was removed by evaporation using a rotary evaporator. The residue was freeze dried and kept at −20 °C for future analysis.

### 3.4. Total Phenolic Content

The total phenolic content of ginger extracts was determined using the Folin-Ciocalteu reagent method with slight modifications. Briefly, ginger crude extract (1 mL, 1 mg/mL in ethanol) or a standard solution of gallic acid (20–100 mg/mL) was mixed with distilled water (9 mL). For the assay, 10-fold diluted Folin-Ciocalteu reagent (1 mL) was added and the mixture was gently shaken. After 10 min, Na_2_CO_3_ solution (1 mL, 7%) was added to the mixture and the total volume was made up to 25 mL by adding distilled water. followed by incubation for 90 min in the dark. The absorbance was determined against a blank at 760 nm using UV-visible spectroscopy. A gallic acid monohydrate standard (>99%; CAS Registry number 5995-86-8) was used to prepare a calibration curve (R^2^ = 0.988). Results were expressed as milligram gallic acid equivalents (GAE)/g DM.

### 3.5. Total Flavonoid Content

Total flavonoid content was measured using the aluminum chloride colorimetric method. In brief, rhizome crude extract (50 µL, 1 mg/mL ethanol) or standard solution of quercetin (20–100 mg/mL) were mixed with NaNO_2_ solution (0.3 mL, 5%); after 6 min incubation at 25 °C, 0.3 mL AlCl_3_ solution (10%) was added. The mixture was mixed well, and allowed to stand for another 6 min. Immediately after that, 1 M NaOH solution (0.2 mL) was added to each extract and incubated for 10 min at room temperature. Absorbance was determined at 510 nm against a blank. Quercetin dihydrate standard (>98%; CAS Registry number 6151-25-3) was used to prepare a calibration curve. Resu.lts were expressed as milligram quercetin equivalents (QE)/g DM.

### 3.6. Identification and Separation of Flavonoids and Phenolic Acids

The chromatographic separation of the flavonoids and phenolic acids was performed using ultra-high performance liquid chr.omatography (Model 1200, Agilent, Santa Clara, CA, USA). The flavonoids and phenolic acids were separated on an Agilent C18 (5 μm of pa.rticle size, 4.6 × 250 mm) reversed-phase column by gradient elution using 0.03 M orthophosphoric acid (A) and HPLC grade methanol (B). The gradient profile was: 0 min 40% B, 10 min 100% B, 15 min 100% B, and 20 min 40% B. The detector wavelengths were set at 280 and 360 nm. The flow rate and injection volume was 1 mL/min and 10 µL, respectively. Column temperature was set at 35 °C. Identification of the compounds was achieved by comparison of retention times with standards, UV spectra and UV absorbance ratios after co-injection of samples and standards. Standards including (−)-epicatechin (≥98%; CAS Registry number 490-46-0), (+)-catechin hydrate (≥98%; CAS Registry number 225937-10-0), kaempferol (≥97%; CAS Registry number 520-18-3), quercetin dihydrate (≥98%; CAS Registry number 6151-25-3), rutin hydrate (≥94%; CAS Registry number 207671-50-9), gallic acid monohydrate (>99%; CAS Registry number 5995-86-8), ferulic acid (>98%; CAS Registry number 1135-24-6), *trans-*cinnamic acid (≥98%; CAS Registry number 140-10-3), tannic acid (>99%; CAS Registry number 1401-55-4) and syringic acid (≥98.0%; CAS Registry number 530-57-4) were purchased from Sigma-Aldrich, city, Malaysia.

### 3.7. UHPLC Analysis of 6- and 8-gingerols and shogaols

The Agilent Model 1200 UHPLC system was used. The 6-gingerol and 6-shogaol were separated on an Agilent C18 (4.6 × 250 mm, 5 μm) reversed-phase column by gradient elution using: (A) water and (B) acetonitrile. The detector wavelength was set at 280 nm. The flow rate and injection volume were 1 mL/min and 20 µL, respectively. Identification of the compounds was achieved by comparison of retention times with standards (6-gingerol and 6-shogaol), UV spectra and UV absorbance ratios after co-injection of samples and standards. 6-Gingerol (≥98%, CAS Registry number: 23513-14-6), 6-shogaol (≥90%, CAS Registry number: 555-66-8), 8-gingerol (≥95%, CAS Registry number: 23513-08-8), were purchased from Sigma-Aldrich, Selangor, Malaysia and 8-shogaol (≥98%, CAS Registry number: 36700-45-5) was purchased from Chem Faces, Wuhan, China. System suitability requirements: at least five replicate injections of 6-and 8-gingerol and shogaol were performed; the requirements of the system suitability parameters are: (1) symmetry factor is not more than 1.5 (2) percentage of relative standard deviation of the retention time for 6-gingerol and 6-shogaol standards is not more than 2.0%.

### 3.8. Evaluation of Antioxidant Activity

#### 3.8.1. 1,1-Diphenyl-2-picrylhydrazyl (DPPH) Assay

A 100 µM methanolic solution of DPPH was freshly prepared (away from light) and 3 mL of this solution was gently mixed with 3 mL of ginger extract. All solutions were incubated at 25 °C for 30 min in darkness. The absorbance of the test and standard solutions was recorded against a blank (methanol and DPPH solution without sample) at 517 nm. Gallic acid and quercetin were used as the standard controls. The percent of inhibition was calculated using the following formula:

% inhibition = [(A − B)/A)] × 100
(2)

where A is absorbance of control; B is absorbance of sample.

#### 3.8.2. Ferric Reducing Antioxidant Potential (FRAP) Assay

FRAP reagent: 2.5 mL FeCl_3_ (20 mmol/L); 2.5 mL (10 mmol/L) 2,4,6-tripyridyl-S-triazine (TPTZ), and 25 mL acetate buffer (pH = 3.6, 0.3 mol/L) were gently mixed and incubated in a water bath at 37 °C for 20 min (with no light). Ginger extracts (0.2 mL) were dissolved in the FRAP reagent (2.0 mL) and made up to 10 mL. The mixture was then incubated at 25 °C for 30 min in a water bath. The absorbance of the solution (blue colour) was read against the blank (acetate buffer) at 593 nm. A standard curve was prepared using concentrations of 100–1000 mM of FeSO_4_ × 7 H_2_O. The results are expressed in μM of Fe (II)/g DM.

### 3.9. Enzyme Extraction

Dried rhizome powder (50 mg) was mixed with sodium phosphate buffer (50 mL, 10 nM, pH 6.5) and gently homogenised. Solutions were centrifuged at 12,000× *g* for 30 min at 4 °C. The supernatant was separated and ammonium sulphate (10 mL, 99%) was added and then centrifuged at 12,000× *g* for 30 min at 4 °C. The precipitate was dissolved in phosphate buffer (5 mL, pH 6.5) and kept at 4 °C for 24 h.

#### Polyphenol Oxidase Activity (PPO)

A total of 800 µL of catechol (50 mM) was mixed with 50 mM phosphate buffer (pH 6.5), then enzyme extract (200 µL) was mixed with this reaction solution and incubated at 37 °C for 2 h. A sample of 1 mL of substrate solution was used as a control. Absorbance of solutions were measured at 420 nm using a spectrophotometer [37]. Differences in the absorbance of the sample and control at 420 nm was calculated as PPO activity and expressed as U/mg DM.

## 4. Conclusions

The results of this study refute the theory that cool drying methods (freeze drying) are better than hot drying methods (oven drying) in order to maintain the quality of the herbal material. According to our findings, it could be dependent on the plant species, the portion of the herb that is used, or the type of metabolites. For example, in the present study, the highest content of 6- and 8-gingerol was observed in the freeze drying method, while the highest content of 6- and 8-shogaol was observed in the vacuum oven drying method. This supports the idea that 6- and 8-gingerol are sensitive to temperature and could be converted to 6- and 8-shogaol at higher temperature. The findings of the present study indicate that the vacuum oven drying method at 45 °C results in significantly higher content of phytochemicals and may be a suitable method for the drying and preservation of bioactive compounds in *Z. officinale* var. *rubrum*. Drying enhanced the phytochemical constituents of rhizomes by stopping PPO activity compared to fresh samples that had higher PPO activity. Antioxidant potential of *Z. officinale* var. *rubrum* rhizome is attributed mostly to quercetin, 6-, and 8-shogaol content and these phytochemicals can be used as indicators in future studies. Based on the results of the present study, it can be concluded that vacuum oven dried ginger rhizomes can be utilized as an ingredient for the development of value-added food products, because they have high content of phytochemicals with valuable antioxidant potential. Future study is recommended for the optimization of the vacuum oven drying method for this plant.

## Figures and Tables

**Table 1:** Effect of different drying methods on TPC, TFC, 6- and 8-gingerol and shogaol content.

Drying Method	TPC	TFC	6-gingerol	8-gingerol	6-shogaol	8-shogaol
	(mg GAE/g DM)	(mg QE/g DM)	(mg/g DM)	(mg/g DM)	(mg/g DM)	(mg/g DM)
Shade drying	10.77 ± 2.37 ^c^	4.93 ± 0.64 ^c^	3.79 ± 0.52 ^c^	2.84 ± 0.31 ^b^	4.21 ± 0.36 ^c^	3.80 ± 0.27 ^c^
Vacuum oven drying	18.44 ± 3.44 ^a^	8.27 ± 0.81 ^a^	4.61 ± 0.41 ^b^	3.42 ± 0.28 ^a^	7.49 ± 0.62 ^a^	5.73 ± 0.41 ^a^
Freeze drying	13.49 ± 2.07 ^b^	6.44 ± 0.56 ^b^	5.82 ± 0.48 ^a^	3.58 ± 0.41 ^a^	4.66 ± 0.36 ^b^	4.19 ± 0.30 ^b^
Fresh	7.58 ± 0.84 ^d^	3.65 ± 0.47 ^d^	3.14 ± 0.32 ^d^	2.50 ± 0.17 ^b^	2.20 ± 0.21 ^d^	2.12 ± 0.14 ^d^

Data are means of triplicate measurements ± standard deviation. Means not sharing a common single letter in each column for each measurement were significantly different at *p* < 0.05.

**Table 2:** Effect of different drying methods on individual flavonoids and phenolic acids content identified from *Z. officinale* var. *rubrum* rhizome extract.

Drying Method	Epicatechin	Catechin	Kaempferol	Quercetin	Rutin	Gallic Acid	Ferulic Acid	Cinnamic Acid	Tannic Acid	Syringic Acid
Shade drying	0.323 ± 0.027 ^c^	0.643 ± 0.062 ^c^	0.931 ± 0.077 ^c^	0.993 ± 0.079 ^c^	0.471 ± 0.037 ^c^	0.386 ± 0.024 ^b^	0.252 ± 0.012 ^c^	0.174 ± 0.012 ^c^	0.271 ± 0.010 ^c^	0.120 ± 0.008 ^c^
Vacuum oven drying	0.397 ± 0.033 ^a^	0.829 ± 0.059 ^a^	1.091 ± 0.064 ^b^	1.391 ± 0.092 ^a^	0.586 ± 0.049 ^a^	0.442 ± 0.045 ^a^	0.393 ± 0.019 ^a^	0.322 ± 0.026 ^a^	0.319 ± 0.030 ^b^	0.201 ± 0.012 ^b^
Freeze drying	0.355 ± 0.031 ^b^	0.742 ± 0.061 ^b^	1.204 ± 0.095 ^a^	1.281 ± 0.088 ^b^	0.513 ± 0.041 ^b^	0.441 ± 0.036 ^a^	0.327 ± 0.024 ^b^	0.214 ± 0.019 ^b^	0.348 ± 0.029 ^a^	0.228 ± 0.016 ^a^
Fresh	0.211 ± 0.037 ^d^	0.517 ± 0.067 ^d^	0.72 ± 0.058 ^d^	0.527 ± 0.048 ^d^	0.257 ± 0.037 ^d^	0.211 ± 0.017 ^c^	0.167 ± 0.011 ^d^	ND	0.147 ± 0.011 ^d^	ND

Data are means of triplicate measurements ± standard deviation. Means not sharing a common single letter in each column for each measurement were significantly different at *p* < 0.05. ND: not detected. Unit: mg/g DM.

**Table 3:** Antioxidant activities of *Z. officinale* var. *rubrum* Theilade rhizome dried with different methods.

Drying Methods and Positive Controls	DPPH (%)	FRAP (μM of Fe (II)/g)	IC_50_ (μg/mL)
Shade drying	37.64 ± 2.44 ^e^	471.8 ± 18.27 ^e^	34.8 ± 1.27 ^b^
Vacuum oven drying	52.9 ± 3.78 ^c^	566.5 ± 21.60 ^c^	27.2 ± 1.19 ^d^
Freeze drying	48.3 ± 3.17 ^d^	527.1 ± 20.47 ^d^	29.1 ± 1.44 ^c^
Fresh	22.5 ± 2.58 ^f^	348.8 ± 16.42 ^f^	42.5 ± 1.62 ^a^
Quercetin	82.46 ± 4.29 ^a^	890.4 ± 24.16 ^a^	15.9 ± 1.07 ^f^
Gallic acid	68.71 ± 4.11 ^b^	647.1 ± 22.18 ^b^	24.3 ± 1.19 ^e^

Data are means of triplicate measurements ± standard deviation. Means not sharing a common single letter in each column for each measurement were significantly different at *p* < 0.05.

**Table 4:** Correlation analysis between identified phytochemicals and PPO activity with antioxidant activities of *Z. officinale* var. *rubrum* Theilade.

Phytochemicals	DPPH	FRAP
6-gingerol	0.914 **	0.886 **
8-gingerol	0.906 **	0.894 **
6-shogaol	0.921 **	0.942 **
8-shogaol	0.813 **	0.944 **
Quercetin	0.940 **	0.916 **
Rutin	0.730 *	0.815 **
Catechin	0.872 **	0.783 *
Epicatechin	0.786 *	0.772 *
Kaempferol	0.882 **	0.847 **
Gallic acid	0.847 **	0.844 **
Tannic acid	0.752 *	0.811 **
Cinnamic acid	0.829 **	0.766 *
Ferulic acid	0.820 **	0.759 *
Syringic acid	0.773 *	0.741 *
PPO activity	−0.911 **	−0.884 **

* and ** represent significant at *p* < 0.05 and 0.01 percent respectively.

## Abbreviations

The following abbreviations are used in this manuscript:

DPPH	1,1-diphenyl-2-picrylhydrazyl
FRAP	ferric reducing antioxidant potential
PPO	polyphenol oxidase
TFC	total flavonoid content
TPC	total phenolic content
UHPLC	ultra-high performance liquid chromatography
